# Sources and Types of Sexual Information Used by Adolescents: A Systematic Literature Review

**DOI:** 10.3390/healthcare12222291

**Published:** 2024-11-16

**Authors:** Sofia Silva, Joana Romão, Catarina Braz Ferreira, Patrícia Figueiredo, Eduarda Ramião, Ricardo Barroso

**Affiliations:** 1 School of Human and Social Sciences, University of Trás-os-Montes and Alto Douro, 5000-801 Vila Real, Portugal; asofiasilva@utad.pt (S.S.); joanamromao@sapo.pt (J.R.); acferreira@utad.pt (C.B.F.); 2 HEI-Lab: Digital Human-Environment Interaction Labs, Lusófona University, 1749-024 Lisboa, Portugal; patriciac.silva.figueiredo@gmail.com; 3 Faculty of Psychology and Education Sciences, University of Porto, 4200-135 Porto, Portugal; eduarda_ramiao@hotmail.com

**Keywords:** sexuality, sources of information, sexual health, sexuality exploration, adolescents

## Abstract

Background/Objectives: Sexuality is part of everyone’s life, especially during adolescence, when young people are discovering themselves and experiencing several changes. Adolescents need to be informed about their sexuality, so they seek and receive information about it from a variety of sources and on different topics. This systematic review aimed to synthesize studies that explored the sources adolescents use and topics they search for. Methods: Quantitative, qualitative, and mixed articles published in Portuguese, Spanish, or English and with respondents aged between 12 and 20 years were included in this study. To fulfill the proposed objectives, three online databases (EBSCOhost, PubMed, and Web of Science) were used, including a total of 48 studies. Results: The most common sources of information are family, friends, school, and the Internet, depending mainly on the reliability of the source and the anonymity when using it. The main topics adolescents search for are sexual intercourse, contraception, relationships, and LGBT issues, since these are less addressed topics. Conclusions: These results are useful in a practical way as a basis for the development of sex education programs that correspond to the needs of adolescents, since they allow us to know which information they are looking for and the sources from which they can receive it.

## 1. Introduction

Adolescence is a critical phase for the development of sexual identity, understanding, and behavior, in which individuals undergo significant physical, emotional, and cognitive changes [1]. According to Csikszentmihalyi [2] and modern society, adolescence is often categorized within the 10–20 age range, characterized by individual experiences of sexual feelings. During this developmental stage, adolescents demonstrate curiosity about their bodies, relationships, and the broader concept of sexuality [3,4,5], and thus, the information they receive can significantly impact their sexual health, decision-making, and overall well-being [6,7]. According to the Social Learning Theory [8], which proposes that people can learn new behaviors by observing others, where people influence and, in turn, are influenced by the world around them, adolescents can learn new behaviors depending on the type of content and source of information they seek to understand sexuality.

Adolescents obtain information related to sexual education from different sources, each of which can vary in accuracy, reliability, and impact. These sources can be divided into categories that compile trustworthy information and those that do not. On the one hand, trustworthy sources can include school-based sexual education programs, healthcare providers, and educational materials specifically designed to inform and guide adolescents [9]. These sources are typically structured, evidence-based, and intended to offer comprehensive and accurate information about topics such as anatomy, reproductive health, contraception, consent, and healthy relationships [6,10].

On the other hand, those that are considered unreliable sources of sexual information can be diverse and often reflect the cultural, social, and technological landscape in which adolescents are embedded. These can include topics such as peer discussions, family conversations, media exposure (e.g., television, movies, and music), and, increasingly, digital platforms like social media, websites (e.g., pornography), and online forums [1,9,11]. While these sources can offer valuable and different perspectives and peer support, they are also prone to disseminating misinformation, societal biases, and incomplete narratives about sexuality.

Understanding the types and sources of sexual information available to adolescents that have an influence in shaping their behavior is essential for developing effective sexual education strategies. By critically evaluating the strengths and limitations of these sources, educators, parents, and healthcare professionals can better ensure that adolescents receive informed and healthy support in navigating their sexual development [4]. This systematic literature review aims to comprehensively analyze and synthesize the existing scientific evidence on the types and sources of sexual information used by adolescents to acquire knowledge about sexuality, following the research question, “What topics do young people want to know about and what sources do they turn to?” In this sense, this study intends to gather resources and offer recommendations for enhancing sexual education strategies within the adolescent population for more educated decision-making and healthy sexuality.

## 2. Methods

The current systematic literature review was conducted according to the Preferred Reporting Items for Systematic Reviews and Meta-Analysis (PRISMA) guidelines, which aim to ensure the transparency of systematic reviews, reducing possible biases in their structure and interpretation. The PRISMA guidelines promote the quality of systematic reviews by evaluating them following the defined structure and format, which allows the reader to have an easier reading experience [12].

### 2.1. Search and Study Selection Strategy

Studies were identified by searching multiple databases, including EBSCOhost, Web of Science, and PubMed, until 4 July 2024. The search string was used for the title and abstract (“sex* education” OR “sexual health” OR “sexual health education” OR “sex*” OR “sex* behavior”) AND (“sources” OR “sources of information” OR “sources of knowledge” OR “access to information” OR “information resources” OR “information behavior” OR “information seek” OR “types of information” OR “search information” OR “information needs”).

### 2.2. Inclusion and Exclusion Criteria

Two independent reviewers performed study selection according to the PRISMA guidelines to reduce the likelihood of errors in study classification and selection [12]. According to the objectives of this systematic review, the following inclusion criteria were established: (a) empirical quantitative and qualitative studies; (b) studies published in English, Portuguese, or Spanish; (c) studies with an adolescent population aged between 12 and 20 years, which better fits adolescents within the school context; and (d) studies mentioning types and/or sources of sexual information. The exclusion criteria were as follows: (a) studies that were missing data—studies that did not describe the direction of the results; (b) case studies, theoretical studies, systematic reviews, and meta-analyses; and (c) studies with samples younger than 12 and/or older than 20 years. The agreement index in this systematic review for the study selection process was assessed with Cohen’s Kappa and revealed a substantial agreement of *K* = 0.78, *p* = 0.001. Disagreements between reviewers were discussed and resolved by consensus.

### 2.3. Identification and Screening

A total of 7481 articles, published between 1970 and 2024, were identified through the databases. Among them, a total of 5007 studies were screened (*n =* 2445 were removed for being duplicates). From their abstracts, 161 articles were retained after eliminating 4875 studies because they (*n =* 2063) included participants younger than 12 or older than 20 years; due to publication type, with 1258 studies that were systematic reviews or meta-analyses; or due to a wrong outcome, with 1554 studies that were not relevant to the topic of this systematic review.

**Figure 1:**
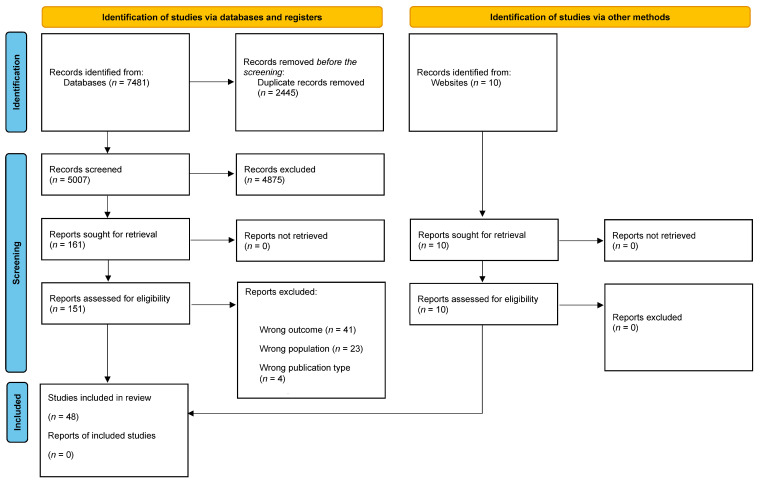
PRISMA 2020 flow diagram of literature review process.

A total of 151 articles were registered for eligibility, 10 of which by hand search, and after full-text analysis, 102 articles were excluded due to wrong outcome (*n* = 41), lack of access (*n* = 35), wrong population (*n* = 23), or wrong publication type (*n* = 4). In total, this systematic review included a total of 48 articles (see Figure 1). Research objectives, methodological aspects, and main findings were extracted from each study.

### 2.4. Quality and Risk Bias of Quantitative and Qualitative Studies

To assess the methodological quality of the studies included in this systematic review, JBI Appraisal Tools were used [13], which provide information that should be included in study reports and standards for good research. Since quantitative and qualitative studies were included, two appraisal tools were used: the JBI-Qualitative Appraisal Instrument and the JBI-Analytical Cross-Sectional Appraisal Checklist. The first consists of 10 items that can be answered as “yes” (Y), “no” (N), “unclear” (U), or “not applicable” (N/A). The second has nine items, and the response system is similar. It was possible to verify that most of the articles meet the criteria presented in the checklist. Given these data, it was decided to include all the articles initially planned (see Table 1 and Table 2).

## 3. Results

**Figure 2:**
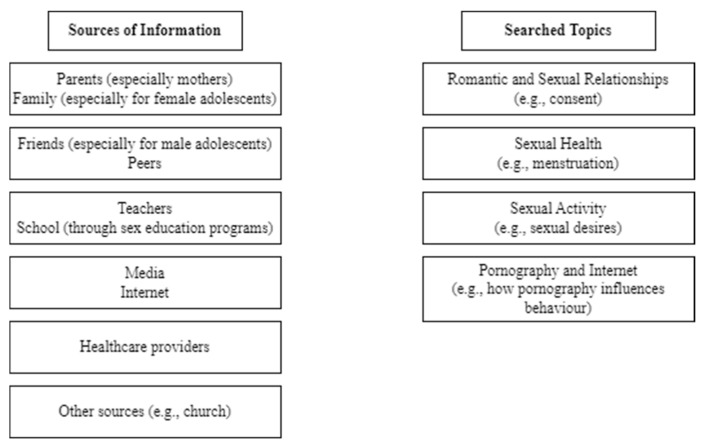
Sources of information and searched topics by adolescents.

The main characteristics and findings of the reviewed studies regarding the sources adolescents relied on to know more about sexuality and the type of content they wished to explore and search for are displayed in Table S1 (Supplementary Materials) and Figure 2.

### 3.1. Study Characteristics

Most studies (*n* = 29) presented a quantitative design [34,35,36,37,38,39,40,41,42,43,44,45,46,47,48,49,50,51,52,53,54,55,56,57,58,59,60,61,62], followed by a qualitative design (*n* = 16; [14,15,16,18,19,20,21,23,24,25,26,27,28,29,31,32,33]), and three used a mixed-methods approach [22,30]. The lowest number of participants in the quantitative studies were 19 [39] and the highest 3180 [42], and the lowest in qualitative studies were 12 participants [16,24] and the highest 128 participants [23]. The participants’ age ranged between 12 years old [30,46,58] and 20 years old [15,32].

### 3.2. Sources of Information Identified by Adolescents

#### 3.2.1. Family

The results showed that adolescents rely on parents to obtain information about sexuality [15,19,21,22,24,25,26,29,35,38,40,41,43,44,45,52,53,54,55,56,57,58,59,60,62]. In particular, mothers were considered trusted sources [19,20,37,44,51,61] more frequently for female adolescents [15,30,46,47,52]. However, fathers were also mentioned in one study [30].

Family was also found to be considered a source of information about sexuality for adolescents [16,20,23,28,31,33,47,50,55,61], identifying aunts [19,29], stepparents [21], grandparents [21], siblings [35,57], and other adult caregivers to be accessible to obtain sexual information [21,41]. In particular, for female adolescents, figures such as mothers, aunts, sisters, and cousins were found to be a great source of information for this population [35].

#### 3.2.2. Friends/Peers

Adolescents mentioned relying on friends [20,22,28,30,31,33,34,35,36,37,39,41,43,44,46,48,50,51,56,57,58,61,62] and peers to obtain information regarding sexuality [15,16,19,23,24,26,27,29,30,33,38,42,45,47,48,50,52,54,55,60].

Specifically, male adolescents tended to obtain more information about sexuality from friends [25] and/or peers [15] compared to females. Also, adolescents who belong in the LGBTQ+ community searched for information about sexuality with peers within the same community [18].

#### 3.2.3. School and Teachers

Within the school context, the results showed that adolescents sought out information about sexuality [16,18,19,24,26,27,30,32,33,34,39,41,42,44,48,50,55,56,59,60,61] through sex education programs [20], as well as with teachers [15,19,21,23,35,37,40,42,43,46,51,52,54,56,58].

#### 3.2.4. Media and the Internet

Overall, media was used to obtain sexual information by youth [19,20,26,29,33,35,41,50,52,54,57,58,60], more specifically, through movies [19,37], television [16,24,37,38,39,61,62], music [37], magazines [25,37,39,43,50], radio [16,39], books [25,39], and newspapers [25].

The internet was also considered a source from which adolescents could access information about sexuality [15,16,20,22,23,25,28,32,34,37,41,42,46,47,48,49,50,51,53,59,61,62]. From the internet, it was possible to find several platforms used by adolescents to inform themselves about sexual content, more specifically, social media [16,56], internet pornography sites [28,31,46,48], and video games [37]. In [15], it was found that male adolescents tended to rely on the internet to obtain sexual information.

Also, in [18], which explored adolescents in the LGBTQ+ community, it was found that adolescents tended to look for sexual information on other online platforms (i.e., Tumblr, blogs, and message boards).

#### 3.2.5. Healthcare Providers

Healthcare providers were also found to be considered a source that adolescents relied on to obtain information about sexuality [21,23,34,36,40,41,49,55], such as centers [35], doctors [18,35,43] or medical professionals [20], community organizations (e.g., [28]), and community-based non-governmental organizations [49].

#### 3.2.6. Other Sources

Some studies (*n* = 3) mentioned the church [16] or priests/pastors [35] as sources that adolescents used to obtain sexual information. Also, adolescents in relationships usually searched for information with their girlfriends/boyfriends [20,41]. In addition, other adults were identified, such as staff of community centers, celebrities, friends of the family, and sources such as condom packaging, community centers, and outreach materials as sources of information about sexuality [20], as well as the Department of Social Services [32].

### 3.3. Type of Information

#### 3.3.1. Relationships

Adolescents desired more information regarding romantic [16,38,39,57] and sexual relationships [23,24,29,31,43,47,48,50,51,57,58], especially about how to initiate a sexual relationship [22], consent [22,24,49,56], and how to manage relationships [30]. In [15], it was found that male adolescents sought more information about relationships. This was also evident in [18], which studied the educational experiences of transgender youth, identifying the need to obtain information regarding relationships (i.e., dealing with rejection and consent). In [26], adolescents mentioned wanting more information regarding how to navigate sexual relationships, how to ensure mutual satisfaction [33], and how to negotiate safe sex practices.

In romantic relationships, adolescents wished and searched for more information regarding having a better preparation for emotional regulation [14] and about the emotional complexities involved in those relationships [33,61], better communication skills to avoid conflict and maintain relationships [14], and on how to maintain healthy relationships through communication and respect [26,28,44,59].

**Communication and Emotions in Relationships.** Studies also found that adolescents wanted to obtain more information related to how to communicate with partners [53] on the topic of sexual health [20], the emotional challenges associated with sexual relationships [27], how to deal with breakups or understanding love and attraction [30], and the emotional aspects of relationships [33]. For girls, it was found that emotional and relational aspects were preferred topics [61].

#### 3.3.2. Sexual Health

**Pregnancy and Contraception.** Regarding sexual health, studies showed topics such as pregnancy [39,49] and its prevention [14,15,23,41,43,44,58] through contraceptive use [19,20,22,24,27,30,32,33,34,36,39,43,46,48,51,52,54,59,61], highlighting condom use [19,20,29,31,36,38,40,41,53] and birth control [38,40]. Adolescents showed interest in obtaining more information on contraceptive use side effects and how to use them correctly [19,55,58]. In [47], female adolescents mentioned seeking information regarding pregnancy, whereas male adolescents tended to look for contraception.

**Menstruation.** Studies identified that adolescents desired to know more about menstruation [39,51,52], especially for female youth [14,15], as well as fetal development and abortion [35].

**Sexual Disease Prevention.** Adolescents showed interest in wanting to know more about how to prevent sexually transmitted diseases (STDs) such as HIV [19] and sexually transmitted infection (STI) prevention [20,23,24,27,29,40,44,46,48,49,50,51,54,55,58,59,61].

**Body Anatomy.** Adolescents sought information about anatomy [14,50], especially girls, who wanted to know more about anatomy and physiology [47].

**Health Services.** Adolescents expressed wanting to obtain information related to where they could access sexual health services [20].

#### 3.3.3. Sexual Activity

Studies found that adolescents wanted more information regarding sexual activity [16,30,36,39,52,54], safe sex practices, [31,44], sexual desires [16], how to avoid sexual coercion and the long-term implications of early sexual activity [19], body changes [39], and sexual behaviors [48]. Likewise, adolescents reported wanting more information regarding consent [20,31,32] and managing the number of sexual partners [20], and female adolescents mentioned wanting to learn more about abstinence [49]. In [14], females in particular desired more information on sexual activity. In [15], male adolescents wanted to know more about sexual performance.

Ref. [18] explored sex education experiences within the LGBTQ+ community, where it was found that adolescents desired to know more about safe sex practices in the community [28] and how to manage gender dysphoria during sexual activity. Also, the same study found that adolescents wanted to gain more information about homosexuality. In the same line, Ref. [28] found that LGBTQ+ adolescents wanted to understand and explore their gender identity and sexual orientation.

Moreover, in [58], adolescents mentioned wanting more information about self-stimulation (i.e., normalcy and health implications of masturbation). Female adolescents in particular sought emotional and relational guidance on the dimensions of sex [54].

#### 3.3.4. Pornography and the Internet

Adolescents also expressed the necessity to learn more about pornography [56], i.e., how pornography influences sexual behavior and how to critically assess online information [53]. Digital literacy was also mentioned by adolescents [59].

## 4. Discussion

Adolescence is a fundamental stage of life characterized by significant changes, especially in sexual development, as adolescents embark on a journey of self-discovery [1]. To become well-adjusted adults with established sexual identities, functions, and abilities, adolescents must successfully shape their understanding and behaviors in the various stages of pubertal and sexual development [63]. Despite the availability of various channels that have sexual information, the accuracy, reliability, and comprehensiveness of these sources can significantly influence the quality of knowledge that adolescents acquire [64]. In this sense, there is a pressing need to explore the variety of sources (e.g., family, peers, digital media) that adolescents use that contribute to their sexual education and to understand which topics they search for and desire to know more about when receiving sexual education. Understanding these factors will help the scientific community in developing accurate and suitable sexual education programs for adolescents. To address these concerns, the present systematic literature review was conducted following the PRISMA guidelines [12], aiming at analyzing the types of sources and information that adolescents seek out to acquire knowledge about sexuality and sexual behavior. A total of 48 articles were included based on the defined inclusion and exclusion criteria.

Studies show that adolescents rely on their family or parents to obtain information about sexuality, being considered the most trustworthy source of information (e.g., [15,35,36,37,38]). Scientific evidence shows that adolescents recognize that sexual education should be begin at home, emphasizing that parent–child communication about sexuality plays a crucial role in leading to a positive influence on adolescents’ choices [36,39,41,43,57,58] and preparing them for adopting good practices regarding their sexual behavior [65]. According to [66], parents should be the main source responsible for providing information on sexual behavior, as they play an important role in their children’s sex education [67]. In certain situations, cultural barriers, limited knowledge, reluctance, and parents’ discomfort in discussing sexual health hinder open communication when topics related to sexuality arise [68]. This also affects their ability to answer their children’s questions on the subject [69], prompting children to seek alternative sources of information.

Adolescents tend to resort to friends or peers to obtain sexual information, as they allow adolescents to talk openly with someone whom they feel comfortable with, considering it a safe space [18,22,27,28,29,31,43,54,70]. This source of information can encourage adolescents to engage in early sexual activity, and it is also associated with risky sexual behavior, e.g., avoidance of condom use [28,29,36,37,41,52,54,60,62].

Previous studies show that teachers are considered a source of information about sexuality as well. According to [71], teachers were the most reliable source for adolescents to discuss sex education. However, adolescents report feeling that teachers are unprepared to provide sex education, are uncomfortable discussing these topics, and even show indifference [72]. As a result, they tend to emphasize abstinence and focus on the negative aspects of sexuality [18,30,39,41,46,54]. Also, the literature recognizes that abstinence is the desirable message for teachers to convey when talking about sex education (e.g., [73,74]).

Nonetheless, school-based sexual health education provides useful information [20,26,31,33,37,43,46,47,48,61,62]. However, these institutions only focus on biological aspects, anatomy, the physical changes that occur during puberty, and abstinence, which gives a poor perspective on sexuality [14,18,26,28,40,44,53,60]. Similar results were also found in the literature since anatomy and the reproductive system were the most discussed topics concerning sex education [65,72].

The media and the internet (e.g., pornography, social media, movies) were also found to be a source of information that adolescents rely on to satisfy their sexual curiosity (e.g., [20,28,48]). Studies suggest that pornography is one of the tools provided by the Internet that is increasingly used by teenagers since it is easily available, inexpensive, and anonymous [18,29,31,46,48,53]. This shift has markedly transformed the way sexual health content is consumed and disseminated among younger audiences, as well as how it influences them, making adolescents susceptible to web-based content and misguidance regarding sexuality, since these sources can be more untrustworthy and adolescents need to have an awareness that some information is too general, impersonal, and unreliable [18,24,38,54]. Therefore, it is important that adolescents can filter the information they receive and recognize its reliability [23,49,62].

Despite having the most reliable information, health professionals were the least cited source of information. These results were not confirmed in the literature, since adolescents point to health professionals as one of the main sources of information on sex education [75,76]. Adolescents do not recognize these professionals as someone they can trust, so they do not turn to them for information until they are older [20,29,32,39,43,49].

Despite increased accessibility and emphasis on information about sexuality, through the present systematic literature review, it is still considered taboo, with prejudices and stereotypes. To better understand the needs of youth about sex-related themes, it is important to understand which information they wish they had received and what they identified as missing. Usually, adolescents have some knowledge about topics such as pregnancy, contraception, and sexually transmitted diseases (STDs), however, they desire more specific information (e.g., pregnancy prevention, contraceptive use and its side effects [19,33]). Adolescents desire to know more about relationships (e.g., romantic and sexual), especially when it comes to consent, emotional regulation skills, communications skills (with their partner [14] and how to avoid sexual coercion [19]), since these topics are not typically explored. Also, studies suggest that adolescents expressed a need for more information about sexual activity (e.g., wanting to talk about experiences [36]), where the lack of sources of information considered reliable (e.g., sexual education programs) that deal with this topic potentially leading to adolescents’ behavior being conditioned by the misleading and unrealistic information they have access to [14,20,23,24,29,31,39,49]. Additionally, new topics have emerged, including LGBTQ+ themes, where adolescents express interest in learning more about LGBTQ+ experiences (e.g., how to engage in safe sex in non-heterosexual relationships [18,28]). Adolescents also seek more information on issues related to pornography, specifically how it can influence their sexual behavior, how to critically evaluate the information it presents, and to interpret the sexual content [53,56]. Even though pornography can answer adolescents’ questions, the information they receive appears to be insufficient, and they may expect pornography to be identical to real-life encounters [77,78].

## 5. Limitations and Future Directions

This systematic review has some limitations. There was a risk of reporting bias due to language limitations, as only English, Portuguese, and Spanish studies were included. This may have limited the diversity of perspectives, as sexuality is influenced by cultural, political, legal, ethical, religious, and philosophical aspects of life. In addition, the terms used to define topics or sources of sexual information may have varied from study to study, which may have resulted in some studies not appearing in the search. It is worth mentioning that since our sample only included 48 studies out of 7481 of the total search, it is difficult to generalize the data for such a sensitive topic; also, studies between 1981 and 2024 were included, so the diversity of information can change over time due to the development of information and communication technologies and access to them. Therefore, it is suggested for future studies to take this into consideration.

Sexuality is still considered a taboo, which may have the effect of young people not being properly informed. Knowing the needs of adolescents and the main sources of information they use is useful for developing future sex education programs. Being aware that adolescents increasingly use the internet, this media could be used as a viable source of information, including the participation of young people in the implementation of online prevention and awareness campaigns, promoting their engagement. Also, as parents have a major role in adolescents’ lives, providing them with strategies and information to talk knowledgeably about sexuality issues, the same can be applied to teachers. The findings suggest a lack of awareness and knowledge, especially within the LGBTQIA+ community; therefore, future research should take into consideration the needs of adolescents and analyze how family dynamics, gender roles, and cultural expectations influence the type and quality of information adolescents receive to adopt inclusive sex education policies.

## 6. Conclusions

Adolescents use a wide range of sources of information and search for various topics; however, it is important to filter that information to offer accurate and reliable knowledge. Receiving information from various sources allows them to access more and deeper information regarding sexual health and what they want specifically. In addition, it enhances the development of a critical perspective on the information and the sources from which it comes. This systematic review gathered scientific literature related to the sources adolescents use to obtain sexual information and their topic preferences, which can expand and enhance the community response to this problem. It is essential to develop strategies to enable parents, peers, teachers, and health professionals to develop the necessary skills to deliver high-quality, needs-based sexual education to young people, allowing for the full development of their sexuality and empowering them to make the healthiest sexual choices.

## Figures and Tables

**Table 1:** Quality and risk of bias of qualitative studies.

Study	Q1	Q2	Q3	Q4	Q5	Q6	Q7	Q8	Q9	Q10	Y	N	U	N/A
Adams & Williams (2011) [14]	Y	Y	Y	Y	Y	N	N	Y	Y	Y	8	2	0	0
Adzovie & Adzovie (2022) [15]	Y	Y	Y	Y	Y	N	Y	Y	N	Y	8	3	0	0
Agbeve et al. (2022) [16]	U	Y	Y	Y	Y	N	N	Y	Y	Y	7	2	1	0
Arbeit et al. (2016) [17]	Y	Y	Y	Y	Y	N	N	Y	Y	Y	8	2	0	0
Bradford et al. (2018) [18]	Y	Y	Y	Y	Y	Y	N	Y	Y	Y	9	1	0	0
Byansi et al. (2023) [19]	Y	Y	Y	Y	Y	N	N	Y	Y	Y	8	2	0	0
Dolcini et al. (2012) [20]	Y	Y	Y	Y	Y	N	N	Y	Y	Y	8	2	0	0
Gabster et al. (2022) [21]	Y	Y	Y	Y	Y	N	N	Y	Y	Y	8	2	0	0
Hammer et al. (2010) [22]	Y	Y	Y	Y	Y	N	Y	Y	Y	Y	9	1	0	0
Jayasundara (2021) [23]	Y	Y	Y	Y	Y	N	N	Y	U	Y	7	2	1	0
Lesta et al. (2008) [24]	Y	Y	Y	Y	Y	N	N	Y	U	Y	7	2	1	0
Low et al. (2007) [25]	Y	Y	Y	Y	Y	Y	Y	Y	U	Y	9	0	1	0
McKee et al. (2015) [26]	Y	Y	Y	Y	Y	Y	N	Y	U	Y	8	1	1	0
McKellar et al. (2019) [27]	Y	Y	Y	Y	Y	N	N	Y	Y	Y	8	2	0	0
Naser et al. (2020) [28]	Y	Y	Y	Y	Y	N	N	Y	Y	Y	8	2	0	0
Nobelius et al. (2010) [29]	Y	Y	Y	Y	Y	Y	Y	Y	Y	Y	10	0	0	0
Powell (2008) [30]	Y	Y	Y	Y	Y	Y	N	Y	Y	Y	9	1	0	0
Rosengard et al. (2012) [31]	Y	Y	Y	Y	Y	N	Y	Y	Y	Y	9	1	0	0
Ross et al. (2020) [32]	Y	Y	Y	Y	Y	Y	Y	Y	Y	Y	10	0	0	0
Teitelman et al. (2009) [33]	Y	Y	Y	Y	Y	N	Y	Y	Y	Y	9	1	0	0

Note. Q1. Is there congruity between the stated philosophical perspective and the research methodology? Q2. Is there congruity between the research methodology and the research question or objectives? Q3. Is there congruity between the research methodology and the methods used to collect data? Q4. Is there congruity between the research methodology and the representation and analysis of data? Q5. Is there congruity between the research methodology and the interpretation of results? Q6. Is there a statement locating the researcher culturally or theoretically? Q7. Is the influence of the researcher on the research, and vice-versa, addressed? Q8. Are participants, and their voices, adequately represented? Q9. Is the research ethical according to current criteria or, for recent studies, is there evidence of ethical approval by an appropriate body? Q10. Do the conclusions drawn in the research report flow from the analysis, or interpretation, of the data? Y—yes; N—no; U—unclear; N/A—not applicable.

**Table 2:** Quality and risk of bias of quantitative studies.

Study	Q1	Q2	Q3	Q4	Q5	Q6	Q7	Q8	Y	N	U	N/A
Abreu et al. (2022) [34]	N	Y	Y	Y	N	N/A	Y	Y	5	2	0	1
Baird (1993) [35]	Y	Y	Y	Y	N	N/A	Y	Y	6	1	0	1
Berenson et al. (2006) [36]	N	Y	Y	Y	N	N/A	Y	Y	5	2	0	1
Bleakley et al. (2009) [37]	Y	Y	Y	Y	N	N/A	Y	Y	6	1	0	1
Bleakley et al. (2018) [38]	Y	Y	Y	Y	Y	Y	Y	Y	8	0	0	0
DeSantis et al. (1999) [39]	Y	Y	Y	Y	Y	N	Y	Y	7	1	0	0
Donaldson et al. (2013) [40]	Y	Y	Y	Y	Y	N	Y	Y	7	1	0	0
Eversole et al. (2016) [41]	N	Y	Y	Y	Y	Y	Y	Y	7	1	0	0
Fang et al. (2022) [42]	N	U	Y	Y	Y	Y	Y	Y	6	1	1	0
Gil et al. (2001) [43]	N	Y	Y	Y	N	N/A	Y	Y	5	2	0	1
Hammer et al. (2010) [22]	Y	Y	Y	Y	Y	Y	Y	U	7	0	1	0
Hampton et al. (2005) [44]	N	Y	Y	Y	Y	N	Y	Y	6	2	0	0
Handelsman et al. (1987) [45]	Y	Y	Y	Y	N	N/A	Y	Y	6	1	0	1
Hernández et al. (2022) [46]	N	Y	Y	Y	N	N/A	Y	Y	5	2	0	1
Jabareen & Zlotnick (2022) [47]	Y	Y	Y	Y	N	N/A	Y	Y	6	1	0	1
Li et al. (2009) [48]	N	Y	Y	Y	N	N/A	Y	Y	5	2	0	1
Macharia et al. (2021) [49]	Y	Y	Y	Y	N	N/A	Y	U	5	1	1	1
Malek et al. (2010) [50]	N	Y	Y	Y	N	N/A	Y	Y	5	2	0	1
Mataraarachchi et al. (2023) [51]	Y	Y	Y	Y	U	N	Y	Y	6	1	1	0
Mayekiso & Twaise (1993) [52]	N	Y	U	U	N	N/A	Y	U	2	2	3	1
Nelson et al. (2018) [53]	Y	Y	Y	Y	N	N/A	Y	Y	6	1	0	1
Pereira (1993) [54]	N	Y	Y	Y	N	N/A	Y	Y	5	2	0	1
Pistella & Bonati (1998) [55]	Y	Y	Y	Y	N	N/A	Y	Y	6	1	0	1
Powell (2008) [30]	N	Y	Y	Y	N	N/A	Y	Y	5	2	0	1
Roth et al. (2021) [56]	N	Y	Y	U	N	N/A	Y	Y	4	2	1	1
Ruiz-Canela et al. (2012) [57]	N	Y	Y	Y	Y	N	Y	Y	6	2	0	0
Sánchez (2006) [58]	N	Y	Y	Y	N	N/A	Y	Y	5	2	0	1
Scharmanski & Hessling (2022) [59]	Y	Y	Y	U	N	N/A	Y	Y	5	1	1	1
Thornburg (1981) [60]	N	Y	Y	Y	N	N/A	Y	U	4	2	1	1
Vázquez et al. (2020) [61]	N	Y	Y	Y	N	N/A	Y	Y	5	2	0	1
Whitfield et al. (2013) [62]	Y	Y	Y	Y	N	N/A	Y	Y	6	1	0	1

Note. Q1. Were the criteria for inclusion in the sample clearly defined? Q2. Were the study subjects and the setting described in detail? Q3. Was the exposure measured in a valid and reliable way? Q4. Were objective, standard criteria used for measurement of the condition? Q5. Were confounding factors identified? Q6. Were strategies to deal with confounding factors stated? Q7. Were the outcomes measured in a valid and reliable way? Q8. Was appropriate statistical analysis used? Y—yes; N—no; U—unclear; N/A—not applicable.

## Data Availability

Data are contained within the article or Supplementary Materials.

## Supplementary Materials

The following supporting information can be downloaded at: https://www.mdpi.com/article/10.3390/healthcare12222291/s1, Table S1: Summary of the Studies’ Included.
