# Supplementary material for: Sources and Types of Sexual Information Used by Adolescents: A Systematic Literature Review
Source: Healthcare (Basel). 2024 Nov 16;12(22):2291. doi: 10.3390/healthcare12222291 (PMC11593387; doi:10.3390/healthcare12222291)
Supplement: Supplementary file 1 [file healthcare-12-02291-s001.zip › Table_S1.pdf]

Table S1

Summary of the Studies' Included

Author (year)	Study Design	Sample N	Research Objectives	Type of Measure	Main Findings	
					Sources of Information	Topics/Themes
Abreu et al. (2022)	Quantitative	N = 190<br>Male: 54.7%<br>Female: 44.7%<br>Age = 14-19<br>M_{age} = 16.7 years	Improve general knowledge and the correct use of contraceptive methods by adolescents, through an alternative approach to sex education in a school context.	Questionnaire	School<br>Internet<br>Friends<br>Healthcare providers	Contraception
Adams and Williams (2011)	Qualitative	N = 75<br>Male: 46.67%<br>Female: 53.33%<br>Age = 15-17<br>M_{age} = 16.04 years	Needs-based assessment of what youth wish they would have known about romantic and sexual relationships.	Semi-structured interviews	NA	Adolescents desired better preparation for emotion regulation of romantic relationships;<br>Better communication skills to avoid conflict and maintain relationships;<br>Females, in particular, desired more information on sexual activity, pregnancy prevention, and anatomy.
Adzovie and Adzovie (2022)	Qualitative	N = 21<br>Male: 57.14%<br>Female: 42.86%<br>Age = 18-20<br>M_{age}= NA years	Identify trusted sources of sexual and reproductive health information among Ghanaian adolescents and reasoning for choosing specific sources over others	Interviews	Parents (especially mothers)<br>Internet<br>Peers<br>Teachers<br>Males rely on the internet and peers<br>Females were more likely to trust mothers	Male adolescents seek information about sexual performance and relationships;<br>Females tended to seek information about menstruation, pregnancy prevention, and others.
Agbeve et al. (2022)	Qualitative	N = 20<br>(12 adolescents, 8 parents)<br>Age = 16-19<br>M_{age}= NA years	To analyse parents and adolescents' views regarding adolescent sexuality education, in Ghana;<br>To understand how do parents and adolescents perceive the role of sexuality education in shaping adolescents' sexual and reproductive health	Semi-structured interview (one-on-one and group)	Peers<br>Media (i.e., radio and television)<br>Social media<br>Internet<br>School<br>Church<br>Family	Sexual desires<br>Sexual acts<br>Dating<br>Relationships

Table S1. (continued)

Author (year)	Study Design	Sample N	Research Objectives	Type of Measure	Main Findings	
					Sources of Information	Topics/Themes
Baird (1993)	Quantitative	N = 81<br>Male: 29.63%<br>Female: 70.37%<br>Age = 14-19<br>M_{age}= 16.5	Examine attitudes, knowledge, and sources of information of adolescents	Sexuality Questionnaire	Teachers<br>Parents<br>Friends<br>Doctors<br>Health care centers<br>Media<br>Siblings<br>Priest/pastor<br>For girls: mothers, aunts, sisters, cousins, and friends	Females mentioned to wanting to learn more about fetal development and abortion
Berenson et al. (2006)	Quantitative	N = 892<br>Male: 0%<br>Female: 100%<br>Age = 15-18<br>M_{age}= 16.9	Examine the link between sexual behavior and hearing discussions among friends	Self-reported survey	Friends<br>Family	Sexual intercourse<br>Contraceptive use<br>Condom use
Bleakley et al. (2009)	Quantitative	N = 459<br>Male: 40.5%<br>Female: 59.5%<br>Age = 14-16<br>M_{age}= 15 years	Examine how sources of sexual information influence adolescents' behaviour	Sources of Sexual Information Survey	Friends<br>Teachers<br>Mothers<br>Media (i.e., television, movies, music, internet, magazines, and video games)	NA
Bleakley et al. (2018)	Quantitative	N = 1990<br>Male: 51%<br>Female: 49%<br>Age = 14-17<br>M_{age} = 15.6	Identify how do adolescents learn about sexual topics from different sources and how those influence sexual behaviours.	Sources of Information, Sexual Activity and Contraceptive Methods Survey	Parents<br>Peers<br>Media (i.e., television and movies)	Sexual intercourse<br>Condom use<br>Hormonal birth control<br>Romantic relationships
Bradford et al. (2018)	Qualitative	N = 14<br>Male: 5<br>Female: 8<br>Age = 14-18<br>M_{age} = 16.1 years	Explore the sex education experiences of transgender young and summarize their recommendations for transgender-curricula	Semi-structured interviews	Online platforms (i.e., Tumblr, blogs, and message boards)<br>Peers (particularly those within the LGBT community)<br>Doctors<br>School	Information that directly addresses transgender issues (i.e., how to have safe sex, dating, and managing gender dysphoria during sexual activity)<br>Relationships (i.e., deal with rejection and consent)<br>Safe sex practices.

Table S1. (continued)

Author (year)	Study Design	Sample N	Research Objectives	Type of Measure	Main Findings	
					Sources of Information	Topics/Themes
Byansi et al. (2023)	Qualitative	N = 32<br>Male: 0%<br>Female: 100%<br>Age = 14-17<br>M_{age} = NA years	Analyse the sexual health behaviours and sexual literacy of adolescent girls in Ugandan	Semi-structured interviews	Parents (especially, mothers and aunts)<br>Teachers and school<br>Media (i.e., movies, billboards, and advertisements)<br>Peers	Pregnancy prevention methods (i.e., contracpetives, their use and their side effects)<br>Condom use (i.e., how to use them correctly to prevent HIV and STDs)<br>Risk associated with sexual relationships (i.e., how to avoid sexual coercion and long-term implications of early sexual activity)
DeSantis et al. (1999)	Quantitative	N = 19<br>Male: NA<br>Female: NA<br>Age = 13-19<br>M_{age} = 14.5 years	Compare and contrast concepts of sexuality and reproduction between parents and adolescents	Sexuality Questionnaire	Media (i.e., books, magazines, radio, and television)<br>School<br>Friends	Pregnancy and contraception<br>Dating and marital relationships<br>Body changes<br>Menstruation<br>Sexual intercourse
Dolcini et al. (2012)	Qualitative	N = 81<br>Male: 49.38%<br>Female: 50.62%<br>Age = 15-17<br>M_{age} = NA years	Examined sexual health information networks among urban African American youth	Semi-structured interview	Friends<br>Family (especially mothers)<br>Sex education<br>Medical Professional<br>Girlfriend or boyfriend<br>Media<br>Internet<br>Other adults (i.e., staff at community centers, celebreties, teachers, coaches, friends of the family)<br>Other sources (i.e., self, job, outreach materials, condom packaging, community centers)	Contraception<br>STI prevention<br>Sexual health (i.e., practical aspects: how to use condoms, where to access sexual health services)<br>How to communicate with partners about sexual health<br>Sexual assertiveness<br>Consent<br>Managing number of sexual partners
Donaldson et al. (2013)	Quantitative	N = 1901<br>Male: 53.97%<br>Female: 46.03%<br>Age = 15-19<br>M_{age} = NA years	Describe how sexually experienced adolescents receive sexual health information	Sexual health information sources and topics	Parents<br>Teachers<br>Healthcare providers	Birth control<br>Condom use<br>STI/HIV protection

Table S1. (continued)

Author (year)	Study Design	Sample N	Research Objectives	Type of Measure	Main Findings	
					Sources of Information	Topics/Themes
Eversole et al. (2016)	Quantitative	N = 1186<br>Male: 49.8%<br>Female: 50.2%<br>Age = NA<br>M_{age} = 14.2 years	Examine the relation between adolescents' source information and their plan to use condoms	Demographic and Sexual Survey	Parents<br>Another relative<br>School<br>Friends<br>Less common: health care providers<br>Media/internet<br>Boyfriend/Girlfriend	Condom use<br>Pregnancy prevention
Fang et al. (2022)	Quantitative	N = 3180<br>Male: 49.7%<br>Female: 50.3%<br>Age = NA<br>M_{age} = 16.9 years	To investigate the associations between sexual knowledge, access to sex education in school and family contexts and sexual experiences.	Questionnaire	Schools/Teachers<br>Internet<br>Peers	NA
Gabster et al. (2022)	Qualitative	N = 20<br>Male: 50%<br>Female: 50%<br>Age = 14-19<br>M_{age} = NA years	Describe where and from what sources young people learn about sexual and reproductive health, as well as what topics carers talk about with adolescents.	Semi-structured interviews	Teachers<br>Healthcare professionals<br>Parents<br>Stepparents<br>Grandparents<br>Other adult caregivers	NA
Gil et al. (2001)	Quantitative	N = 955<br>Male: 38%<br>Female: 62%<br>Age = NA<br>M_{age} = 16 years	Investigate the sources of sexual information, and preferred topics.	Questionnaire	Friends<br>Magazines<br>Teachers<br>Parents<br>Doctors	Contraception and pregnancy prevention<br>Sexual relationships
Hammer et al. (2010)	Mixed	N = 44<br>Male: 52%<br>Female: 48%<br>Age = 13-18<br>M_{age} = NA years	Evaluate levels of knowledge of sexual health, STIs, pregnancy, and information seeking behavior	Semi-structured interview<br>Discussion guide<br>Base-line Questionnaire	Parents<br>Friends<br>Internet	Contracpetion (i.e., effective contraceptive methods) and Pregnancy Prevention<br>Sexual relationships (i.e., initiation and consent)
Hampton et al. (2005)	Quantitative	N = 2353<br>Male: 50%<br>Female: 50%<br>Age = 15-18<br>M_{age} = 15.6 years (grade 10), 17.6 years (grade 12)	Understand the relationship between parental communication, peer influence and sexual activity	Sexuality Questionnaire	Friends<br>Parents (particularly mothers)<br>Schools	How to maintain healthy relationships (i.e., communication and respect)<br>Pregnancy and STI Prevention (i.e., contraceptive methods, STI prevention)<br>Safe sexual practices

Table S1. (continued)

Author (year)	Study Design	Sample N	Research Objectives	Type of Measure	Main Findings	
					Sources of Information	Topics/Themes
Handelsman et al. (1987)	Quantitative	N = 113<br>Male: 31%<br>Female: 69%<br>Males:<br>Age = 13-19<br>Mage = 15.2 years<br>Females:<br>Age = 13-18<br>Mage = 15.5 years	Understand if positive communication with parents would be related to responsible sexual behavior	Questionnaire	Parents<br>Peer	Sex<br>Contraception
Hernández et al. (2022)	Quantitative	N = 159<br>Age = 12-18<br>M_{age} = NA years	Explore sources of sexual information	Sexuality Questionnaire	Mothers (especially for female adolescents)<br>Internet (e.g., pornography)<br>Friends<br>Teachers	Sexual relationships<br>Contraception<br>Sexually transmitted infections
Jabareen & Zlotnick (2022)	Quantitative	N = 558<br>Male: 50.31%<br>Female: 49.06%<br>No identification: 0.63%<br>Age = 16-18<br>M_{age} = NA years	Determine if sources of sexual knowledge differed by gender	Sexual Knowledge Questionnaire<br>Rosenberg Self-Esteem Scale	Family (mothers and sisters for female participants)<br>Peers<br>Internet	Anatomy (girls)<br>Physiology (girls)<br>Pregnancy (girls)<br>Contraception (boys)
Jayasundara (2021)	Qualitative	N = 128<br>Male: 60.94%<br>Female: 39,06%<br>Age = 17-19<br>M_{age} = 18 years	Determine what factors predict sexual information-seeking behaviour	Interviews	Internet<br>Peers<br>Family<br>Healthcare Providers<br>Teachers	Risk associated with sexual activity (i.e., STI and pregnancy prevention)<br>Sexual relationships and behaviour
Lesta et al. (2008)	Qualitative	N = 12<br>Male: 50%<br>Female: 50%<br>Age = 15-18<br>M_{age} = NA years	Examine the sources and adequacy of the information received by youth from various sources on sexuality and sexual health	Semi-structured discussion guide	Peers<br>Media (i.e., television)<br>School<br>Parents	Contraception (i.e., how to use it effectively)<br>STI Prevention (i.e., particularly beyond HIV/AIDS)<br>Sexual relationships and mutual consent<br>Homosexuality
Li et al. (2009)	Quantitative	N = 2545<br>Male: 33.8%<br>Female: 66.2%<br>Age = NA<br>M_{age} = 15.6 years	Explore adolescents' sexuality related attitudes, behaviours, and sources of information	Health Related Behaviour Questionnaire	Friends and peers<br>School<br>Internet<br>Pornography	Sexual relationships<br>Sexual behaviours<br>Contraception<br>STI prevention

Table S1. (continued)

Author (year)	Study Design	Sample N	Research Objectives	Type of Measure	Main Findings	
					Sources of Information	Topics/Themes
Low et al. (2007)	Qualitative	N = 31<br>Age = 13-17<br>M_{age} = 14.8	Explore adolescents' sexuality, sources of sexual information and how it influenced their attitudes	Semi-structured interviews	Friends (boys)<br>VCDs (video compact disks)<br>Internet<br>Newspaper<br>Books<br>Magazines	NA
Macharia et al. (2021)	Quantitative	N = 133<br>Male: 46%<br>Female: 54%<br>Age = 15-19<br>M_{age} = 17 years	Investigates sources and their limitations and explore the role of technology	WHO questions on adolescent sexual health	Healthcare services (older adolescents)<br>Parents (younger adolescents)<br>Community-based non-governmental organizations<br>Internet	Abstinence, unwanted pregnancies, STIs and drugs (Younger adolescents, especially girls)<br>Sexual relationships (i.e., consent and mutual respect)
Malek et al. (2010)	Quantitative	N = 2600<br>Male: NA<br>Female: NA<br>Age = 15-18<br>M_{age} = NA years	Study students' sources of sexual knowledge acquisition	Self-reported Questionnaire	Friends and Peers<br>Media (i.e., magazine, books) and electronic media (CDs, internet)<br>Family<br>School	Basic sexual knowledge (i.e., anatomy, reproduction, and STI prevention)<br>Practical adivce (i.e., sexual behaviour and relationships)
Mataraarachchi et al. (2023)	Quantitative	N = 810<br>Male: 0%<br>Female: 100%<br>Age = 14-19<br>M_{age}= 15.3 years	To assess the sexual and reproductive health issues discussed between Sri Lankan female adolescents and their mothers.	Questionnaire	Mothers<br>Teachers<br>Friends<br>Internet	Menstrual health<br>Personal boundaries<br>Sexual health risks (i.e., STIs, contraception)<br>Sexual Relationships
Mayekiso & Twaise (1993)	Quantitative	N = 50<br>Male: 0%<br>Female: 100%<br>Age = 15-16<br>M_{age} = NA years	Examined the extent of parental involvement in imparting sexual knowledge to adolescents	Knowledge and Perception of Sexuality Questionnaire	Peers<br>Parents (i.e., mothers with daughters)<br>Teachers<br>Media	Menstrual health<br>Contraception<br>Sexual activity
McKee et al. (2015)	Qualitative	N = 89<br>Male: NA<br>Female: NA<br>Age = 14-16<br>M_{age} = NA years	Find what young people know about sex and how they talk about that.	Semi-structured interview	Schools<br>Parents<br>Peers<br>Media	Pratical sexual health information (i.e., navigate sexual relationships, how to ensure mutual satisfaction, and how to negotiate safe sex practices)<br>Emotional and relational aspects of sex (i.e., how to communicate desires and boundaries with a partner)

Table S1. (continued)

Author (year)	Study Design	Sample N	Research Objectives	Type of Measure	Main Findings	
					Sources of Information	Topics/Themes
McKellar et al. (2019)	Qualitative	N = 33<br>Male: 0%<br>Female: 100%<br>Age = 13-14<br>M_{age} = 13.6 years	Understand how youth think about sexuality and the ways in which they engage with information	Paper-based diary	Peers<br>Incidental Media exposure (i.e., posters or television shows)<br>School	Contraception<br>STI Prevention<br>Biological aspects of sex (i.e., desire for clear, factual information)<br>How to navigate the social pressures<br>Emotional challenges associated with sexual relationships
Naser et al. (2020)	Qualitative	N = 17<br>Male: 17.65%<br>Female: 82.35%<br>Age = 14-18<br>M_{age} = 16 years	Describe the experiences of LGBT youth with school sexual education, and how they address their specific sexual health needs	Semi-structure interviews	Internet<br>Community Organizations<br>Friends<br>Family<br>Pornography	Safe sex practices for LGBTQ+ relationships (i.e., how to engage in safe sex in non-heterosexual relationships)<br>Emotional and relational aspects of sex (i.e., how to communicate with partners and navigate sexual relationships)<br>Gender identity and sexual orientation (i.e., understanding and exploring their gender identity and sexual orientation).
Nelson et al. (2018)	Quantitative	N = 207<br>Male: 100% (who are interested in sex with males)<br>Female: 0%<br>Age = 14-17<br>M_{age} = 16 years	Determine where male adolescents who are interested in males receive sexual health information, clarify their preferences, and explore relations with sexual behavior	Standardized Questionnaire	Internet<br>Parents	Practical sexual health (i.e., how to safely and comfortably engage in anal sex, use condoms and lubrication)<br>Communication with sexual partners<br>Pornography (i.e., how pornography influences sexual behaviour and how to critically assess online information)
Nobelius et al. (2010)	Qualitative	N = 31<br>Male: NA<br>Female: NA<br>Age = 13-15<br>M_{age} = NA years	Understand how adolescents receive sexual information and how they would prefer to receive	Unstructured interviews	Ssenga (paternal aunt)<br>Parents<br>Peers<br>Media (i.e., radio, pamphlets)	Sexual relationships (i.e., safe sex)<br>Contraceptive use<br>STI prevention<br>Negotiate condom use
Pereira (1993)	Quantitative	N = 100<br>Male: 50%<br>Female: 50%<br>Age = NA<br>M_{age} = 16 years	Analyze the main sources about sexuality used by youth and what sources they would like to turn to.	Sexuality Questionnaire	Peers<br>Parents<br>Media<br>Teachers	Basic sexual knowledge (i.e., sexual intercourse, contraception, and STIs)<br>Emotional and relational aspects: girls in particular sought guidance on the emotional and relational dimensions of sex.

Table S1. (continued)

Author (year)	Study Design	Sample N	Research Objectives	Type of Measure	Main Findings	
					**Sources of Information**	**Topics/Themes**
Pistella & Bonati (1998)	Quantitative	N = 249<br>Male: 0%<br>Female: 100%<br>Age = 13-19<br>M_{age} = 16.5 years	Examine the sexual topics that adolescents discussed with their parents	Structured Questionnaire	Peers<br>Parents (and Family members)<br>School<br>Healthcare providers	Contraceptive options and use (i.e., contraceptive methods, how to access them, and how to use them effectively)<br>STD prevention
Powell (2008)	Mixed	N = 401<br>Male: 46%<br>Female: 54%<br>Age = 12-19<br>Mage = 13.9 years	Explore how youth gain sexual information and advice	Self-reported questionnaires<br>Interviews	Friends and peers<br>Mothers (for female adoleswcents)<br>Fathers<br>School and Media	Contraception<br>Safe sex practices<br>Managing relationships<br>Emotional support: dealing with breakups or understanding love and attraction
Roth et al. (2021)	Quantitative	N = 1603<br>Male: 26%<br>Female: 70.6%<br>Transgender/gender diverse: 3%<br>Others: 0.4%<br>Age = 12-18<br>M_{age} = 16 years	Better understand adolescents' views and experiences of sexual health education	Survey	School and Teachers<br>Friends<br>Social Media<br>Parents	Gender identity and sexual diversity<br>Consent and decision-making (i.e., how to navigate sexual relationships, understand consent, and make informed decisions)<br>Media representation and pornography (i.e., interpreting sexual content and understand the impact on real-life sexual behaviour)
Rosengard et al. (2012)	Qualitative	N = 69<br>Male:44.93%<br>Female: 55.07%<br>Age = 15-18<br>M_{age} = NA years	Understand family as a information sources and how affects their behavior	Semi-structured Interview	Family<br>Friends<br>Media (pornography)	Practical guidance (i.e., how to engage in safe sexual practices, use of condoms and understanding consent)<br>Balanced information (i.e., positive aspects of sexual relationships)
Ross et al. (2020)	Qualitative	N = 16<br>Male: 0%<br>Female: 100%<br>Age = 18-20<br>M_{age}= 19 years	Explored the experiences of youth in foster care related to sexual health	Interviews	Internet<br>School<br>Department of Social Services	Comprehensive sexual and reproductive health information (i.e., contraception, puberty, and sexual consent)<br>Support and guidance (i.e., supportive and non-judgmental manner)<br>The main reported sources were internet, school, healthcare professionals and sexual abuse.
Ruiz-Canela et al. (2012)	Quanitative	N = 2615<br>Male: 56.14%<br>Female: 43.86%<br>Age = 13-19<br>M_{age} = 15 years	Analyze the influence of sexuality knowledge and the first intercourse	Questionnaire	Parents<br>Friends<br>Siblings<br>Media	Sexual relationships (i.e., when to start and how to navigate romantic and sexual relationships)

Table S1. (continued)

Author (year)	Study Design	Sample N	Research Objectives	Type of Measure	Main Findings	
					Sources of Information	Topics/Themes
Sánchez (2006)	Quantitative	N = 501<br>Male: 54.69%<br>Female: 45.30%<br>Age = 12-16<br>M_{age} = 13.3 years	Describe sexual behaviours, their sources of information, sexual knowledge and requests for information.	Sources of information and sexual knowledge survey	Parents<br>Friends<br>Media<br>Teachers	Sexual relationships (i.e., understand the emotional and physical aspect of sexual relationships)<br>Contraceptive methods (i.e., how to use contraceptives effectively to prevent unwanted pregnancies and STIs)<br>Self-stimulation (i.e., normalcy and health implications of masturbation)
Scharmanski and Hessling (2022)	Quantitative	N = 3556<br>Male: 43.08%<br>Female: 56.92%<br>Age = 14-17<br>M_{age} = NA years	To investigate where young Germans get their knowledge about sexuality and contraception, as well as which people, institutions and media contribute to the development of this knowledge and what role the internet plays.	Survey	School<br>Parents<br>Internet	Contraception and safe sex (i.e., contraception methods, safe sex practices, and how to prevent STIs)<br>Relationships and emotions (i.e., managing relationships, understanding sexual consent and navigating the emotional aspects of sexual relationships)<br>Digital literacy
Teitelman et al. (2009)	Qualitative	N = 33<br>Male: 0%<br>Female: 100%<br>Age = 14-18<br>M_{age} = NA years	Exploring perspectives about the social context in which they learn about sexuality	Interviews	Family<br>Friends/Peers<br>School<br>Media	Protection methods<br>Emotional aspects of relationships<br>How to navigate sexual pleasure<br>Guidance on managing relationships and understanding the emotional complexities involved
Thornburg (1981a)	Quantitative	N = 1152<br>Male: 47.05%<br>Female: 52.95%<br>Age = 14-18<br>Mage = NA years	Identify initial sources of information on twelve sexual concepts	Sources of Information Open-ended Questionnaire	Parents<br>Peers<br>Schools<br>Media	NA
Vázquez et al. (2020)	Quantitative	N = 1635<br>Male: NA<br>Female: NA<br>Age = 12-18<br>M_{age} = NA years	Explore the sources of sexual information	Self-reported Questionnaire	Friends<br>Internet/TV<br>Family (i.e., specially mothers)<br>School	Sexual practices (i.e., contraception and protection against STIs)<br>Emotional and relational aspects (especially girls; i.e., relationships and sexual pleasure)
Whitfield et al. (2013)	Quantitative	N = 2036<br>Male: NA<br>Female: NA<br>Age = 13-16<br>M_{age} = NA years	Identify sources of information used by youth to understand adolescents' practices as they develop a sexual health knowledge	Sexual Attitudes and Lifestyle Survey	Friends<br>TV<br>Parent/caregivers<br>Internet	

Note. NA = Not Applicable; STD = Sexually Transmitted Disease; LGBTQ+ = Lesbian, Gay, Bisexual, Transgender, Questioning, Intersex, Two Spirit, and Queer; STI = Sexually Transmitted Infections; LGBT = Lesbian, Gay, Bisexual, Transgender
